# Sleep apnoea is common in severe peripheral arterial disease

**DOI:** 10.1371/journal.pone.0181733

**Published:** 2017-07-31

**Authors:** Nadjib Schahab, Sarah Sudan, Christian Schaefer, Vedat Tiyerili, Martin Steinmetz, Georg Nickenig, Dirk Skowasch, Carmen Pizarro

**Affiliations:** University Hospital Bonn, Department of Internal Medicine II, Cardiology, Pneumology and Angiology, Bonn, Germany; Spectrum Health, UNITED STATES

## Abstract

**Background:**

Atherosclerotic conditions have been demonstrated to be associated with sleep- disordered breathing (SDB). Peripheral arterial disease (PAD) represents severe atherosclerosis with a high mortality. In early stages of PAD a substantial prevalence of sleep apnoea has already been shown. Here, we sought to determine the frequency of undiagnosed sleep apnoea in a homogeneous group of advanced PAD patients undergoing percutaneous revascularization.

**Methods:**

59 consecutive patients (mean age: 71.1 ± 9.8 years, 67.8% males) with PAD in Fontaine stages IIb-IV that underwent percutaneous transluminal angioplasty at our department were enrolled for pre-procedural polygraphy.

**Results:**

Patients appertained to Fontaine clinical stage IIb, III and IV in 54.2%, 23.8% and 22.% of cases, respectively, and were principally intervened for femoropopliteal occlusive disease (71.2% of total study population). Polygraphy revealed sleep apnoea in 48 out of 59 patients (81.4%), of whom 60.4% offered a primarily obstructive-driven genesis. Among those patients with polygraphically confirmed sleep apnoea, mean apnoea hypopnoea index (AHI) and mean oxygen desaturation index (ODI) averaged 28.2 ± 19.5/h and 26.7 ± 18.8/h, respectively. 18 patients even offered an AHI ≥30/h that is indicative of severe sleep apnoea. For obstructive-driven apnoeic events, AHI correlated significantly with PAD severity stages (p = 0.042).

**Conclusions:**

In our PAD collective, sleep apnoea was frequent and obstructive sleep apnoea´s severity correlated with PAD severity stages. Long-term results regarding the vasoprotective impact of CPAP treatment on PAD course remains to be determined.

## Introduction

Obstructive sleep apnoea (OSA) is defined by recurrent episodes of complete or partial collapse of the upper airway [1,2] and subsequent occurrence of apnoea and hypopnoea resulting in gas exchange impairment [3]. Its acute and chronic adverse effects cause oxyhemoglobin desaturation, fluctuations in blood pressure and heart rate, sympathetic activation, increased intrathoracic pressure, systemic inflammation and endothelial dysfunction, the latter predisposing to the development and progression of atherosclerosis [4,5]. The prevalence of OSA as well of sleeping disorderd breathing (SDB) in general increases with age. It is estimated that among the 50–70 year old unselected male population up to 17% suffer from mild to severe SDB, in patients with coronary artery disease (CAD) the prevalence of SDB raises even up to 50% [6,7].

Peripheral artery disease (PAD)—as part of a global vascular problem of diffuse atherosclerosis- represents an underdiagnosed manifestation of atherosclerosis [8]. It exhibits a worldwide prevalence of 2.5% among individuals 60 years and younger and it increases with advanced age until a prevalence of 18.8% among those aged 70 years and older [9]. Beside the correlation between age and PAD, established risk factors for PAD development and progression are congruent to that in CAD: cigarette smoking, arterial hypertension, diabetes mellitus and dyslipidaemia [10]. The impact of OSA on PAD has been investigated by Utriainen et al. in patients with advanced PAD. Due to the study concept, only patients undergoing operative revascularization were examined. In summary, Utriainen et al. detected a substantial OSA prevalence of 85% in an 82 participants comprising study cohort [11]. Consistently, we have already extended these findings to early PAD stages and have ascertained a notable frequency of 78% in sleep disorders [12].

In order to further elucidate the link between sleep related breathing disorders and advanced PAD, the present prospective study examined the frequency of previously undiagnosed SDB and its clinical correlations in patients undergoing percutaneous transluminal angioplasty (PTA) for advanced PAD in Fontaine stages IIb to IV.

## Methods

### Study population

A total of 59 consecutive patients aged ≥18 years with severe PAD IIb to IV (Fontaine) hospitalized for elective PTA at the Department of Angiology (University Hospital of Bonn, Germany) between January 2016 and July 2016 were included in this prospective cohort trial. Based on a sample size of 60 patients we expected to be able to estimate a rate of 70–80% sleep apnoea with a 95% confidence interval (CI) ± 15%. Using standardized questionnaires we assessed cardiovascular risk factors and concomitant medication. Completive appraisal of presented medical reports were utilized for the assessment of comorbidities such as e.g. CAD. Exclusion criteria comprised pre-diagnosed SDB, advanced congestive heart failure (NYHA III-IV), severe valvular heart disease, atrial fibrillation, end-stage renal disease and drug-resistant arterial hypertension. Individuals with early stages of PAD (Fontaine stage I, IIa, ankle brachial index (ABI) ≥0.9) as well as patients with an ABI >1.3 that is associated with mediasclerosis were excluded from study participation. Especially four patients with already diagnosed and treated SDB were not study enrolled.

Informed written consent was obtained from all patients. The study was approved by the local ethic committee of the faculty of medicine of the University of Bonn (Germany) and conducted in accordance with the Declaration of Helsinki.

### Echocardiography

To determine left ventricular systolic and diastolic function as well as valvular status transthoracic echocardiography was executed 4 weeks before sleep apnoea screening. Left ventricular ejection fraction (LVEF) was measured according to the modified Simpson’s method using apical four- and two-chamber views. Blood flow doppler and tissue doppler were used to assess diastolic function.

### Angiological diagnostics

#### Clinical staging

Non-invasive angiological examinations were performed in conformity with the current ESC guidelines on diagnosis and treatment of PAD [13]. Clinical staging was consistent with Fontaine classification, with stage I defining asymptomatic course, stage IIa and IIb comprehending claudication at a walking distance exceeding or below 200 m, respectively, stage III describing ischaemic rest pain and stage IV including a necrotic and/ or gangrenous process.

#### Ankle brachial index (ABI)

Patients were placed in a supine position, and brachial and ankle systolic pressures were obtained. The protocol involved taking routine bilateral upper extremity pressure readings. The cuff was placed on the lower right calf. Doppler was placed behind the medial malleolus to obtain the posterior tibial (PT) systolic pressure and then placed on the top of the foot to obtain the dorsalis pedis (DP) systolic pressure. ABI was calculated by dividing the highest ankle systolic pressure (DP or PT) by the highest systolic pressure from either arm. The process was then repeated on the left ankle.

ABI values ≤ 0.9 are pathological. In diabetic patients with heavily calcified vessels, the arteries are frequently non-compressible. It results in an artificially elevated ankle pressure, offering ABI values >1.3, which can cause underestimation of disease severity.

#### Duplex ultrasound

Additional duplex ultrasound, conducted by versed angiologists, was used for completion of ABI results and enabled assessment of the atherosclerotic plaque

#### Angiography and percutaneous revascularization

The first step during an angioplasty procedure was an angiography. After the angiography one of the following interventional procedures was performed by an experienced interventionist, as indicated: balloon angioplasty alone and balloon angioplasty with a drug-eluting balloon (DEB) or with stent insertion.

Discharge medication included aspirin and four weeks of clopidogrel in case of angioplasty with a DEB or stent insertion.

### Nocturnal SDB screening

All participants underwent an overnight SDB screening by a portable study unit using Embletta MPR^®^ attached as a belt to the patients abdominal surface the night prior to angiography. A continuous recording of respiration and oxyhemoglobin enabled the evaluation of sleep fragmentation and desaturation. Episodes of disordered breathing were categorized as apnoea or hypopnoea, and as either obstructive, if the airflow criteria were met and thoracic and abdominal effort was documented, or central, without any respiratory effort. Apnoea was defined as a complete cessation of airflow or a >90% reduction in airflow from baseline for at least 10 seconds. Hypopnoea was defined as a reduction in airflow of ≥50% with an oxygen saturation decrease of ≥3%. Mixed apnoeic events, defined as absence of nasal airflow associated with a lack of respiratory effort before re-uptaking inspiratory effort in the second portion of the event, were classified as a result of upper-airway instability and thus of obstructive nature [14].

According to the current American Academy of Sleep Medicine manual for scoring respiratory events in sleep [15], patients with an apnoea-hypopnoea index (AHI), defined as the averaged frequency of apnoea and hypopnoea events per hour of sleep, <5/h were considered not to have SDB. Depending on the number of events per hour SDB was classified into mild (AHI: 5.0–14.9 events/ h), moderate (AHI: 15.0–29.9 events/h) and severe (AHI ≥30 events/h) sleep apnoea. For measuring daytime sleepiness we used the Epworth sleepiness scale (ESS), a self-rating questionnaire. An ESS score >9, along with an AHI >5/h, indicated SDB and pronounced daytime symptomatology [16].

The oxygen desaturation index (ODI) was defined as the number of oxygen level drops ≥3% from baseline per hour. In addition, also mean and minimal oxygen saturation, average pulse frequency and snoring proportion were recorded.

### Statistical analysis

Descriptive variables were expressed as absolute numbers and percentages or as mean ± standard deviation (SD). For continuous and normally distributed data, unpaired t-tests were used. For categorical parameters, Pearson’s Chi-squared test was employed. Correlation between numerical data was calculated using Spearmans’ rank correlation. Ordinal variables were analysed by the Kruskal-Wallis and Mann-Whitney-Wilcoxon non-parametric test. A simple correction for multiple pairwise comparison was possible by replacing the Mann-Whitney p-value by the corresponding Kruskal-Wallis p-value in the case that the Mann-Whitney p-value was smaller. In our data we found low evidence for a correlation betweenIn our data we found low evidence for a correlation between age and AHI. So we did no use age as a correction factor for all other analyses regarding AHI. Statistical analyses were performed by use of SPSS Statistics 23 software (IBM, Armonk, NY, USA).

## Results

### Demographic characteristics

A total of 59 patients with angiologically proven severe PAD assigned to overnight sleep apnoea screening were finally analysed. Baseline characteristics and clinical data are displayed in Table 1. The mean age of the included patients was 71.1 ± 9.8 years, 67.8% were male. With a mean body-ass index (BMI) of 27.7 ± 4.3 kg/m^2^ patients were slightly overweight. Continued nicotine consumption was documented in 67.8% of the entire study population. Other established cardiovascular risk factors as diabetes mellitus, arterial hypertension and hyperlipidaemia were present in 33.9%, 96.6% and 57.6% of cases, respectively. Antiplatelet agent and statin therapy was employed in 100% and 96.6% of study population, respectively. CAD was present in 53.3% (8/15) of patients without sleep apnoea and in 61.4% (27/44) of patients exhibiting SDB. Sleep apnoea was not affected by CAD status (p = 0.726). Mean C-reactive protein (CRP) value in patients with OSA was 9.3 mg/l (± 11.0 mg/dl) and did not differ significantly from that obtained in patients without OSA (7.8 mg/l ± 7.33 mg/dl, p = 0.545).

**Table 1:** Demographic and clinical data of the study population at baseline.

	All patients	Fontaine stage IIb	Fontaine stage III	Fontaine stage IV
**Number (%)**	59 (100)	32 (54.2)	14 (23.7)	13 (22.0)
**Male, n (%)**	40 (67.8)	23 (71.9)	8 (57.1)	9 (69.2)
**Age in years**	71.1 ± 9.8	68.4 ± 9.3	71.71 ± 9.6	77.2 ± 8.8
**Body mass index (kg/m^2^)**	27.7 ± 4.3	27.3 ± 4.3	28.2 ± 4.1	28.2 ± 4.5
**NYHA class I, n (%)**	21 (35.6)	12 (37.5)	6 (42.9)	3 (23.1)
**NYHA class II, n (%)**	14 (23.7)	7 (21.9)	3 (21.4)	4 (30.8)
**Systolic blood pressure (mmHg)**	150.1±21.5	151.6 ± 24.7	151.2 ± 21.7	145.8 ± 11.4
***CVRF*** ^a^, ***n (%)***				
**Continued nicotine consumption**	40 (67.8)	22 (68.8)	9 (64.3)	9 (69.2)
**Arterial hypertension**	57 (96.6)	31 (96.9)	13 (92.9)	13 (100)
**Dyslipidaemia**	34 (57.6)	19 (59.4)	6 (42.9)	9 (69.2)
**Diabetes mellitus**	20 (33.9)	7 (21.9)	5 (35.7)	8 (61.5)
***Medication before any intervention*, *n (%)***				
**Antiplatelet agent**	59 (100)	32 (100)	14 (100)	13 (100)
**Beta-blocker**	38 (64.4)	21 (46.9)	9 (64.3)	8 (61.5)
**ACE-inhibitor**	26 (44.1)	15 (47.7)	6 (42.9)	5 (38.5)
**AT1-inhibitor**	20 (33.9)	11 (34.4)	5 (35.7)	4 (30.8)
**Calcium antagonist**	22 (37.3)	11 (34.4)	8 (57.1)	3 (23.1)
**Diuretic**	41 (69.5)	20 (62.5)	11 (78.6)	8 (61.5)
**Statin**	57 (96.6)	31 (96.9)	14 (100)	12 (92.3)
**Digitalis**	1 (1.7)	1 (3.1)	0	0
**Oral antidiabetic**	8 (13.6)	4 (12.5)	2 (14.3)	2 (15.4)
**Insulin**	12 (20.3)	4 (12.5)	3 (21.4)	5 (38.5)
***ABI*** ^b^				
**- Index extremity pre** ^c^	0.60 ± 0.15	0.67 ± 0.14	0.53 ± 0.09	0.51 ± 0.16
***ABI values*, *n (%)***				
** - mild (0.7–0.9)**	19 (32.2)	16 (50)	0	3 (23.1)
** - moderate (0.5–0.69)**	26 (44.1)	13 (40.6)	11 (78.6)	2 (15.4)
** - severe (<0.5)**	14 (23.7)	3 (9.4)	3 (21.4)	8 (61.5)
***Echocardiography***				
**LVEF** ^d^ **in %**	54.8 ± 16.4	53.4 ± 19.0	60.4 ± 9.9	52.4 ± 13.9
**Diastolic dysfunction, n (%)**				
**- no dysfunction**	29 (49.2)	16 (50.0)	5 (35.7)	8 (61.5)
**- °I**	21 (35.6)	12 (37.5)	6 (42.9)	3 (23.1)
**- °II**	9 (15.3)	4 (12.5)	3 (21.4)	2 (15.4)
**-°III**	0	0	0	0

^a^ cardiovascular risk factors;

^b^ ankle brachial index;

^c^ pre-procedural;

^d^ left ventricular ejection fraction.

Data are presented as total number and percentage, mean ± SD

### Angiological characteristics

54.2% of all patients were found to be in Fontaine stage IIb, 23.8% were in Fontaine stage III and 22.0% in Fontaine stage IV. Mean preprocedural ABI was 0.59 (± 0.17) for the treated leg and 0.82 (± 0.24) for the contralateral leg. 33 of 59 patients (55.9%) exhibited bilateral limb disease. For percutaneous revascularization drug eluting balloon was used in 38.1%, stent was implanted in 61.9%. In 71.2% of cases, femoro-popliteal arteries were treated. 15 of 59 patients (25.5%) exhibited a multi-level PAD.

### Frequency of sleep-disordered breathing

SDB results are summarized in Table 2. Nocturnal screening identified a total frequency of sleep apnoea of 81.4% (48/59 patients) with a mean AHI among SDB-patients of 28.2/h (± 19.5/h).

**Table 2:** Sleep variables according to severity and type of sleep apnoea.

Cut-off SDB ^a^ -type	All patients	AHI 5.00–14.90			AHI 15.00–29.90			AHI ≥30.00		
		OSA ^b^	CSA ^c^	all SDB	OSA	CSA	all SDB	OSA	CSA	all SDB
Subjects n (%)	59 (100)	14 (23.7)	3 (5.2)	17 (28.9)	8 (13.5)	5 (8.5)	13 (22.0)	7 (11.9)	11 (18.6)	18 (30.5)
**Sleep study analysis**										
AHI ^d^, events/ h	21.9 ± 20.0	9.7 ± 2.5	10.1 ± 3.6	9.5 ± 2.7	20.8 ± 4.0	22.5 ± 4.0	21.5 ± 4.0	44.9 ± 10.8	48.6 ± 19.8	47.0 ± 16.1
Nocturnal oxygen saturation										
Mean, %	91.1 ± 2.9	91.1 ± 3.5	91.9 ± 3.4	91.3 ± 3.2	91.1 ± 3.2	90.6 ± 0.8	90.0 ± 2.4	90.5 ± 2.5	89.7 ± 3.2	90.0 ± 2.8
Minimal, %	80.2 ± 9.1	84.2 ± 5.2	84.3 ± 7.5	83.9 ± 5.4	81.0 ± 7.3	76.0 ± 6.7	78.9 ± 7.2	72.3 ± 11.5	73.8 ± 10.9	73.2 ± 10.8
ODI ^e^, events/h	20.9 ± 19.1	11.4 ± 5.13	10.8 ± 6.4	11.0 ± 5.1	22.9 ± 10.6	21.7 ± 10.2	22.4 ± 9.9	45.7 ± 14.2	38.2 ± 22.7	41.5 ± 19.2
Longest apnoea duration, s	41.2 ± 23.2	50.7 ± 7.0	22.7 ± 6.6	42.9 ± 31.4	57.2 ± 25.8	45.8 ± 9.5	52.4 ± 20.8	50.9 ± 20.6	40.1 ± 8.8	44.9 ± 15.7
Nocturnal heart rate, bpm ^f^	72.2 ±10.5	67.4 ± 8.5	69.5 ± 10.8	67.1 ± 8.8	68.1 ± 11.1	77.8 ± 16.7	74.2 ± 13.8	74.7 ± 8.1	69.9 ± 9.2	78.9 ± 8.5
Snoring proportion, %	12.9 ± 15.6	7.6 ± 8.6	3.8 ± 5.2	6.6 ± 7.7	7.3 ± 11.8	11.1 ± 10.5	8.9 ± 11.0	41.4 ± 16.1	9.2 ± 1.8	21.8 ± 20.5
Mean record time, h	6.8 ± 1.5	5.9 ± 2.1	7.2 ± 0.6	6.2 ± 1.9	7.2 ± 1.0	7.2 ± 2.9	6.5 ± 2.1	6.7 ± 1.1	7.2 ± 0.569	7.1 ± 0.9

^a^ sleep disordered breathing;

^b^ obstructive sleep apnoea;

^c^ central sleep apnoea;

^d^ apnoea hypopnoea index;

^e^ oxygen desaturation index;

^f^ beats per minute

Data are presented as total number and percentage, mean ± SD

Within this collective, sleep apnoea was mild (AHI: 5.0–14.9/h) in 35.4% (17/48 patients), moderate (AHI: 15–29.9/h) in 27.1% (13/48 patients) and severe (AHI ≥30/h) in 37.5% (18/48 patients). Mean ODI was 26.7/h ± 18.8/h and was significantly correlated with the severity stages of sleep apnoea (p <0.001, Spearman’s rho: 0.91). Furthermore, daytime sleepiness—as measured by ESS—correlated significantly with the AHI (p = 0.003, Spearman’s rho: 0.47): the percentage of patients exhibiting a score >9, which is indicative of excessive sleepiness, was 37.3% (n = 22). 29 patients (60.4%) presented predominantly obstructive episodes with a mean AHI of 23.5/h (± 16.5/h) compared to 19 patients (39.6%) who showed central-driven apnoeic episodes with a mean AHI of 35.0/h (± 21.9/h). With advanced severity degree of sleep apnoea central driven events predominated (p = 0.024).

Presence of bilateral limb disease did neither influence SDB, nor OSA occurrence (p = 0.58 and p = 0.69, respectively).

**Fig 1:**
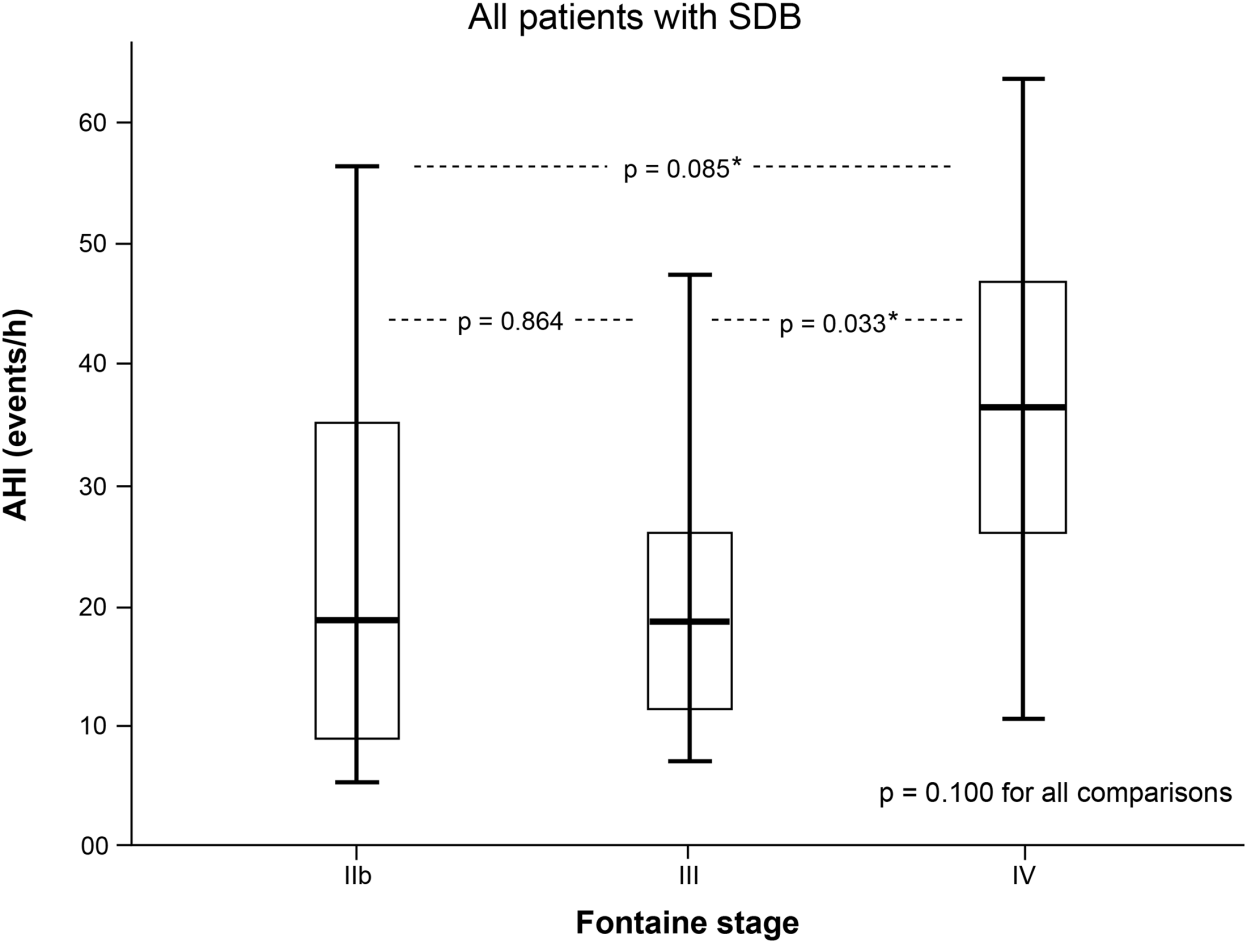
Sleep disordered breathing (SDB) measured by apnoea-hypopnoea index (AHI) and stratified by Fontaine PAD stages. * to be corrected to 0.100 due to multiple testing.

**Fig 2:**
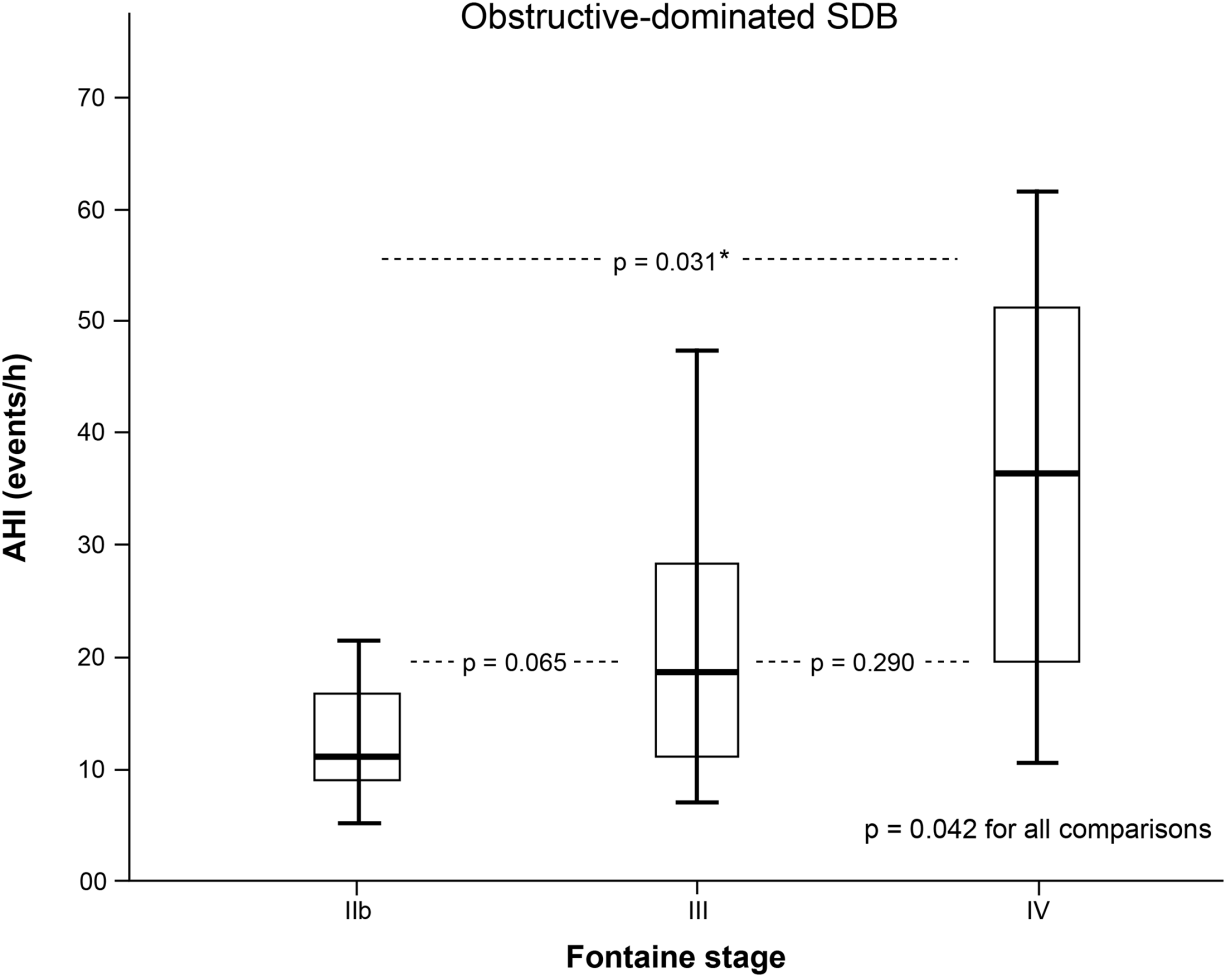
Obstructive-dominated sleep disordered breathing (SDB) measured by apnoea-hypopnoea index (AHI) and stratified by Fontaine PAD stages. * to be corrected to 0.042 due to multiple testing.

**Fig 3:**
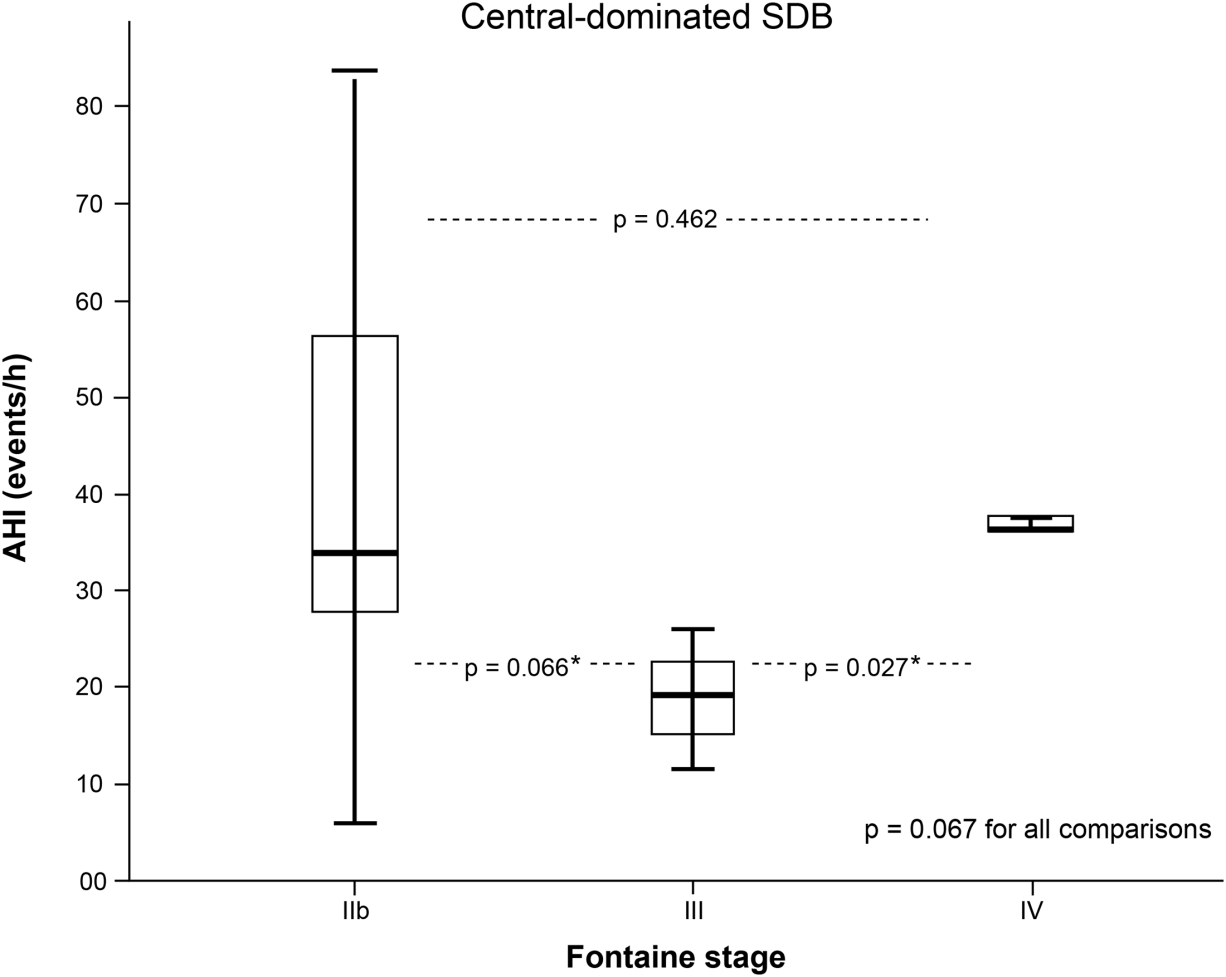
Central-dominated sleep disordered breathing (SDB) measured by apnoea-hypopnoea index (AHI) and stratified by Fontaine PAD stages. * to be corrected to 0.067 due to multiple testing.

**Fig 4:**
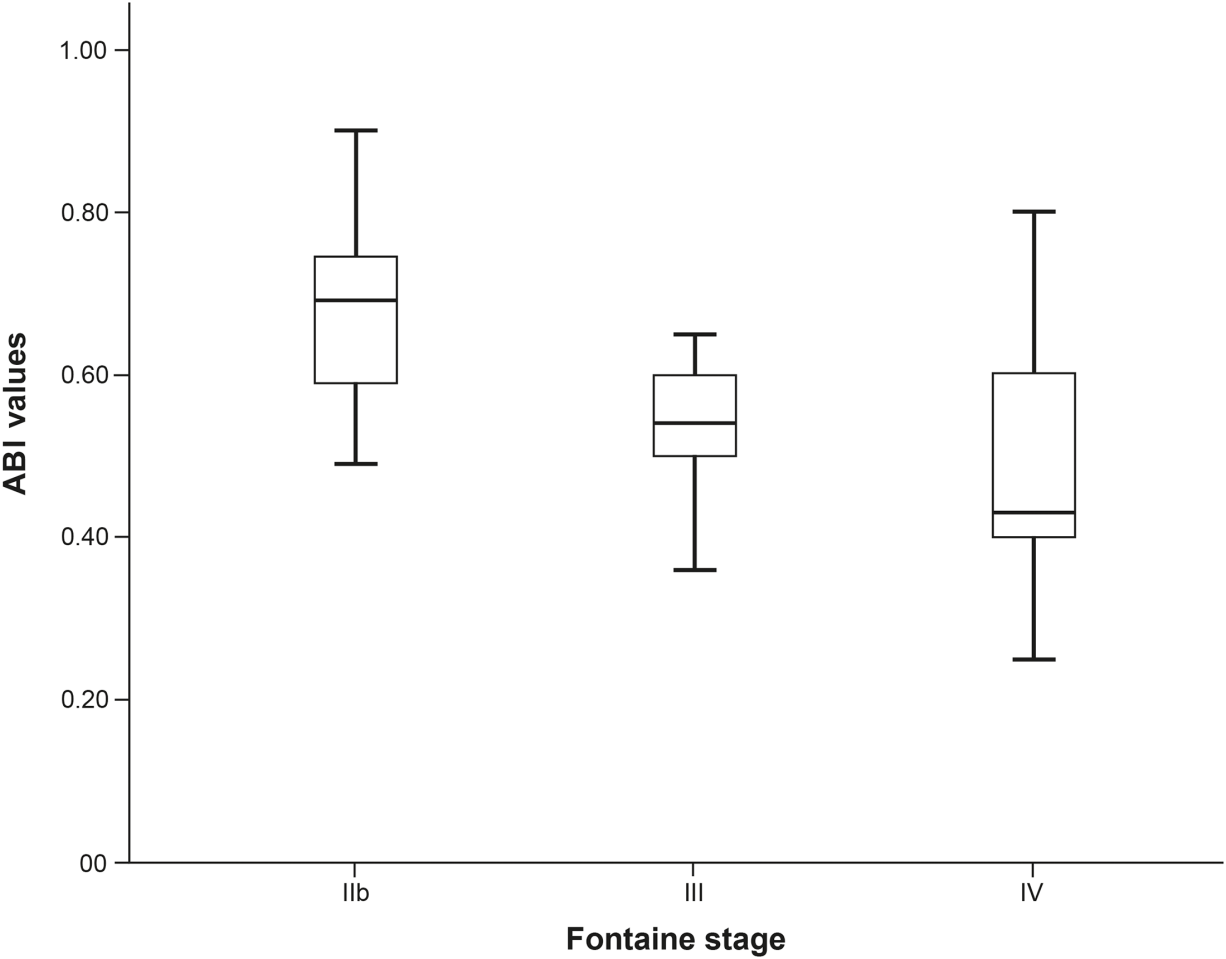
Ankle brachial index (ABI) values stratified by PAD severity stages (p < 0.001).

Results obtained by distribution analysis of AHI over Fontaine stages are displayed in Figs 1–3. AHI values in patients with OSA increased significantly with advanced Fontaine stage (p = 0.042; Fig 2), while in central-driven apnoea we found no association between AHI values and Fontaine stages (p = 0.067; Fig 3). Moreover we evidenced an inverse correlation between ABI and Fontaine stage (p <0.001, Fig 4).

We found 13 patients in Fontaine stage IV of whom 8 patients (61.5%) exhibited a diabetes mellitus. As compared to the totality of Fontaine stage IIb and III patients, Fontaine IV patients offered a significantly higher diabetes mellitus rate (p = 0.014) and were significantly older (p = 0.007) (Table 1).

**Fig 5:**
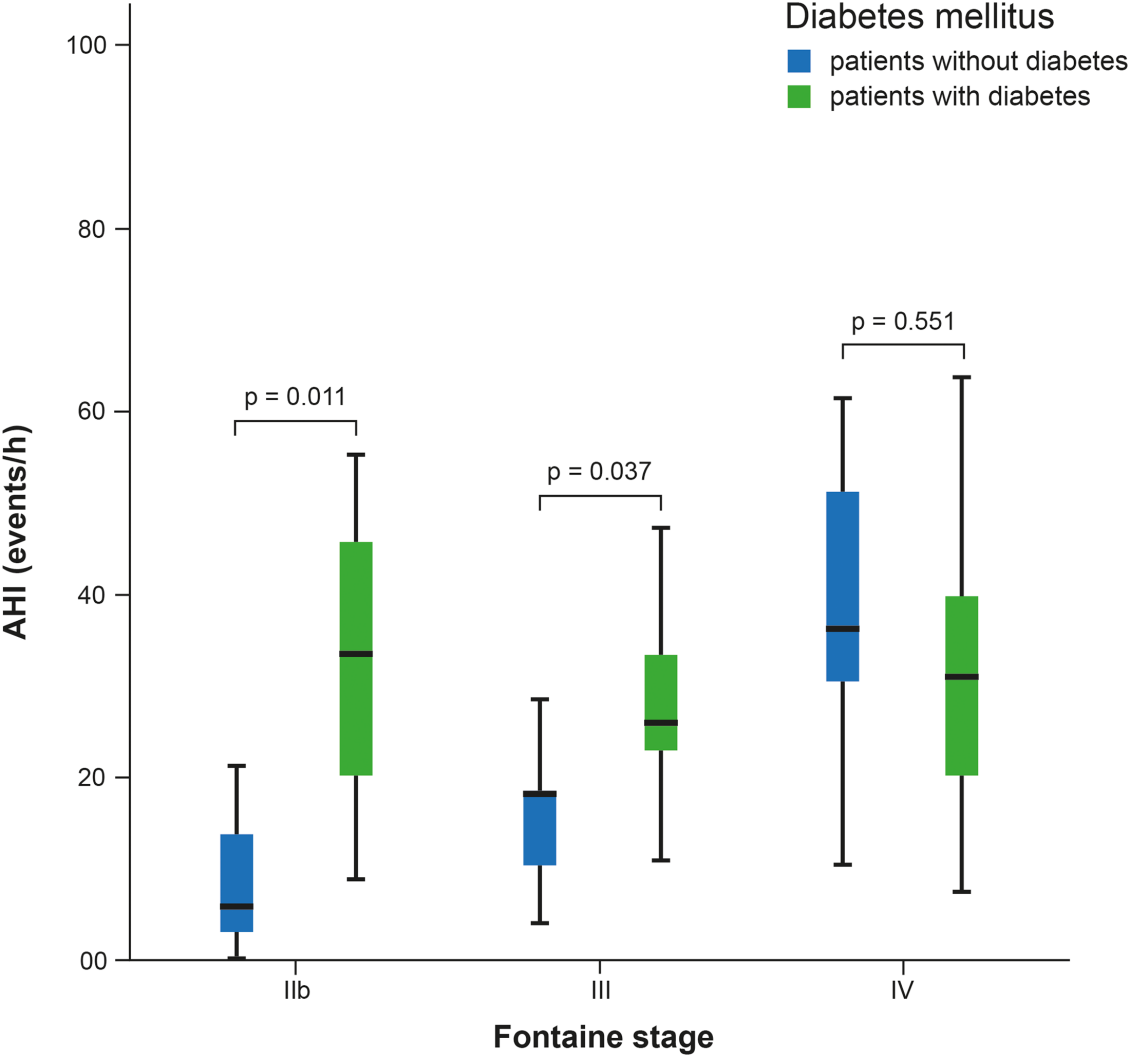
Distribution of AHI depending on diabetes mellitus as a function underlying Fontaine stages.

To further investigate the association between AHI, PAD and diabetes mellitus we performed a two-factorial ANOVA. Analysis indicated a trend towards a higher prevalence of diabetes mellitus with increasing AHI depending on PAD stage (p = 0.075). Fig 5 displays the impact of diabetes mellitus on the AHI, as a function of the underlying Fontaine stage: while in Fontaine stages IIb and III the presence of diabetes mellitus was associated with a significant higher AHI (p = 0.011 and p = 0.037, respectively), in Fontaine stage IV patients this association was absent (p = 0.551).

## Discussion

To the best of our knowledge, this is the first study investigating the frequency of previously undiagnosed sleep-disordered breathing in patients with peripheral artery disease undergoing percutaneous transluminal angioplasty.

The main findings are:

1. There is a high frequency of SDB (obstructive as well as central) in the studied PAD population (81.4%) comprising patients with advanced PAD.
2. Obstructive sleep apnoea in patients with PAD showed a significant relationship between the AHI and the severity of PAD. In the subset of OSA patients OSA severity was associated with increasing Fontaine stages.

There have been previous studies detecting an unexpected high frequency of SDB in patients suffering from PAD. Utriainen et al. demonstrated an OSA frequency of 85% in 82 patients undergoing surgical revascularization with a mean AHI of 21.0/h. We have already examined early PAD stages that presented a sleep apnoea frequency of 78% and a mean AHI of 11.8/h [11,12]. With an identified sleep apnoea frequency of 81.4% our current findings are consistent with the aforementioned study data. In order to make a comparison with SDB frequency in the general population, we referred to prior epidemiologic studies. For example, Preppard et al. found a SDB prevalence of 17% among 50–70 year old men and of 9% among 50–70 year old women in a 1520 participants comprising healthy study population, percentages that substantially underrun the presently found frequency [6].

Whereas in our prior study cohort focusing on patients with less severe PAD (Fontaine I-IIb) the mean AHI was 11.8/h [12], in our current study collective including patients with advanced PAD mean AHI was 28.2/h. In the subset of OSA patients, AHI and consequently OSA severity increased with advanced PAD Fontaine stages. This interaction might be indicative of a mutual pathophysiological correlation. Of interest, we didn't find a significant association between AHI and the preprocedural ABI of the treated leg, although we observed a significant difference in ABI values between PAD severity groups (p-value <0.001).

OSA is believed to be a risk factor not only for coronary but also for atherosclerotic manifestation in the non-coronary vascular bed. Several prior studies have shown an association between OSA and cardiovascular events [17]. As expected, our patients presented established classical cardiovascular risk factors. Except for diabetes mellitus that was more frequent in patients with sleep apnoea (p = 0.008), other cardiovascular risk factors (hypertension, smoking habits, obesity and dyslipidemia) didn’t exhibit significant differences in patients with or without SDB.

Accordingly, the high frequency of sleep apnoea seems to be not solely caused by comorbidities and risk factors. Moreover, the degree of systemic inflammation is influenced by the presence of OSA. Sustained sympathetic activation in consequence of hypoxaemia and recurrent arousals actuates a cascade of inflammation by overbalance of vasoconstrictive agents [18]. In keeping with this, augmented vasoconstriction is due to an OSA-activated elevation in plasma endothelin-1 concentration as an effective vasoconstrictor and leads to exaggerated endothelial cell damage and dysfunction [19,20]. Due to increased levels of cytokines and adhesion molecules that attract monocytes to endothelial cells and consequently promote endothelial damage, the atherosclerotic process is initiated [21]. It has been shown that levels of inflammatory markers like CRP and serum amyloid A are significant increased in patients suffering from OSA as compared to healthy controls. Their concentrations vary as a function of OSA severity degree [22,23]. Likewise, CRP in our present study was slightly elevated in patients with OSA but did not significantly vary between groups.

The fact that age and PAD are highly correlated in our data makes it difficult to decide about causal relationships between age, PAD and AHI. Additional linear model fits to the data revealed that we have quite low evident for age to AHI correlation in our data (AHI = 3.88 + 0.26*age, p = 0.338). A linear model for AHI with age as covariate und PAD stage as factor revealed an even lower evidence for an age effect (regression parameter estimate 0.039 [-.52, 0.60] confidence interval, p = 0.89). But this may be a result of age to PAD correlation and a simple shift of variance components in the linear model. Based on these observations we did not correct for age when comparing AHI between PAD groups. Anyway it cannot be solved statistically whether the observed PAD effect on AHI is just an expression of the age differences between the PAD subgroups.

As previously shown, SDB in patients with atherosclerosis mainly occurs as obstructive sleep apnoea [11,12]. In contrast, our current study exhibits an important rate of central driven events (39.6%). These apnoeas are often associated with congestive heart failure (CHF) [24]. In keeping with this, we consciously excluded patients with advanced CHF by performing a preprocedural echocardiography. Nevertheless the presently observed elevated number of central driven events may be deduced from an important presence of mild CHF as 59.3% suffered from NYHA I-II, as well as 50.9% suffered from slight diastolic dysfunction and 28.8% exhibited a reduced LVEF [Table 1].

Observational clinical studies have demonstrated a benefit of continuous positive airway pressure (CPAP) therapy in CAD patients with concomitant SDB that was accompanied by lower rates of cardiovascular complications among patients who are adherent to treatment [25,26,27,28]. However, in contrast to those studies, recent randomized controlled trials couldn’t verify a significant effect of CPAP therapy on the prevention of long-term cardiovascular events, especially in nonsleepy populations [29,30]. The impact of SDB treatment by CPAP therapy on PAD progression, restenosis and prognosis remains to be determined in further studies.

There are several study limitations that should be addressed. First, we did not perform cardiorespiratory polysomnography which still represents the gold standard in patients with suspected sleep apnoea. In a previous study, Embletta^™^ -based cardiorespiratory polygraphy, as used in our study, was compared with polysomnographic findings: comparison of AHI and ODI showed a statistically significant close correlation between the results obtained by polysomnography and polygraphy [31]. Second, the study protocol did not allot a control cohort that would have permitted direct comparison of SDB frequency in PAD patients and healthy participants. However, in order to make a comparison to SDB frequency in the general population we referred to prior epidemiologic studies [6].

In conclusion, our findings demonstrate that there is a high and still under-recognized frequency of SDB in patients with severe PAD with a preponderance of obstructive driven events. AHI may increase with PAD severity stages, but further research is needed to determine if OSA represents an independent risk factor for the onset and progression of PAD.
